# Dogs fed a high protein, low carbohydrate diet have elevated postprandial plasma glucagon and amino acid concentrations and tend to have lower glucose concentrations compared to two different moderate protein, moderate carbohydrate diets

**DOI:** 10.1093/tas/txaf017

**Published:** 2025-02-11

**Authors:** Sydney Banton, Shari Raheb, Pawanpreet Singh, John P Cant, Anna K Shoveller

**Affiliations:** Department of Animal Biosciences, University of Guelph, Guelph, ON N1G 2W1, Canada; Department of Clinical Studies, University of Guelph, Guelph, ON N1G 2W1, Canada; Department of Animal Biosciences, University of Guelph, Guelph, ON N1G 2W1, Canada; Department of Animal Biosciences, University of Guelph, Guelph, ON N1G 2W1, Canada; Department of Animal Biosciences, University of Guelph, Guelph, ON N1G 2W1, Canada

**Keywords:** amino acid, canine, cardiology, glucose, high protein

## Abstract

As dog owners continue to seek to feed their dogs similarly to themselves, there is demand for high protein, low carbohydrate (HPLC) diets. The consumption of HPLC diets can improve glycemic control, similarly to high fiber diets. However, the effects of HPLC and high fiber diets on cardiac function have yet to be evaluated in healthy dogs. The objective of the present study was to investigate the glucose, insulin, glucagon and amino acid (AA) postprandial response and echocardiographic measurements in laboratory-housed, adult large breed dogs fed a commercially available HPLC, a moderate protein, moderate carbohydrate (MPMC), or a commercially available MPMC, high fiber, “metabolic” diet for 42 d. This study was conducted as a 3 × 3 Latin square where dogs received: 1) a commercial HPLC diet (48% of metabolizable energy (ME) from protein, 10% of ME from nitrogen-free extract; NFE), 2) a MPMC diet (28% of ME from protein, 39% of ME from NFE) formulated with the same ingredients as HPLC or 3) a MPMC, high fiber, “metabolic” (MET) diet (30% of ME from protein, 37% of ME from NFE) as a commercial control. An echocardiogram and a 12-h glucose, insulin and glucagon response and 6-h AA meal response were performed on day 42 of feeding. Data were analyzed using proc glimmix in SAS (version 9.4). All echocardiographic parameters remained within a healthy reference range for dogs of this size. Dogs fed HPLC had a larger net area under the curve (NetAUC) for plasma glucagon (P < 0.001) compared to dogs fed MPMC and MET, a smaller NetAUC for glucose: insulin (P = 0.039) compared to dogs fed MPMC but MET was similar to both. Glucose NetAUC tended to be different among treatments (P = 0.057), where dogs fed MPMC had a greater netAUC than dogs fed HPLC and dogs fed MET tended to have a greater netAUC than HPLC. Dogs fed HPLC had greater concentrations of Ile, Leu, Lys, Thr, Tyr and Val over time compared to dogs fed MPMC and MET, and dogs fed MET had greater concentrations of Gln and Met over time compared to dogs fed HPLC and MPMC (P < 0.05). Dogs fed a HPLC diet may have improved glucose uptake compared to dogs fed a MPMC diet. This research provides the first insight into the cardiometabolic health of dogs consuming three diets differing in their protein, carbohydrate and fiber content.

## Teaser

This manuscript describes a metabolic shift in healthy, adult, large breed dogs fed a high protein, low carbohydrate diet towards greater post-meal glucagon and amino acid concentrations and lower blood glucose concentrations. This could have implications for obese or diabetic dogs with poor glucose uptake.

## INTRODUCTION

There is an increasing demand for higher protein diets for our companion animals, with ‘high protein’ being the second most influential claim on pet food among US respondents in 2022 (Wall, 2022). This trend seems to span across both North America and Europe with the average crude protein content of dry dog foods in Europe being 10% higher than the European Pet Food Industry Federation (FEDIAF) minimum guidelines (Kazimierska et al., 2021). High protein, low carbohydrate (HPLC) diets are not a new concept in human nutrition and have been extensively researched. High protein diets are often reported to increase weight loss and decrease body mass index in obese subjects without calorie restriction (Nickols-Richardson et al., 2005; Krebs et al., 2010). In addition, the consumption of HPLC diets can lower fasted and postprandial plasma glucose in subjects with mild, untreated type 2 diabetes (Seino et al., 1983; Gannon & Nuttall, 2004). Similarly, high fiber diets have also been reported to increase weight loss without calorie restriction (Hays et al., 2004) and decrease postprandial glucose concentrations in people with and without type 2 diabetes (Anderson et al., 1995; Chandalia et al., 2000).

High protein and fiber are often used in combination in dog foods intended for weight loss to maintain lean mass, despite energy restriction (Diez et al., 2002; Phungviwatnikul et al., 2022). In addition, high protein and fiber are thought to increase satiety. In support of this, one study reported a lower voluntary food intake in a high protein, high fiber diet 3 h after an energy-restricted meal compared to just a high protein diet and just a high fiber diet in dogs of ideal body weight (BW; Weber et al., 2007). Similarly to humans, high fiber diets are often used to help control type 1 diabetes in dogs, in addition to insulin, and have been reported to lower fasted and postprandial plasma glucose concentrations (Graham et al., 2002). In contrast to humans, dogs do not develop type 2 diabetes but can develop glucose intolerance in relation to obesity (Kaiyala et al., 1999), and as such, are almost always insulin-dependent (Rand et al., 2004).

There is limited and more conflicting data when it comes to high protein diets and their effects on cardiac health. There is some evidence in overweight humans that a high protein, calorie-restricted diet can decrease biomarkers for cardiovascular risk, such as total cholesterol and triacylglycerol concentrations (Clifton et al., 2009). In addition, in overweight patients with type 2 diabetes, a HPLC diet decreased triacylglycerol concentrations and blood pressure as well as improved diastolic function, as measured by mitral valve peak E-wave velocity (von Bibra et al., 2014). However, in mice, a high protein diet led to increases in blood amino acid concentrations, stimulating macrophage mammalian target of rapamycin (mTOR) signaling and atherosclerotic plaques (Zhang et al., 2020). Thus it has been assumed that the beneficial effects of a high protein diet on cardiac health are largely due to the weight loss induced when these diets are energy-restricted. When it comes to fiber, research is generally more conclusive and suggests that increased dietary fiber can lower cardiovascular disease risk in humans, as assessed via population surveys (Liu et al., 2002; Zhang et al., 2022). Furthermore, in mice fed a high fiber diet, left ventricle ejection fraction and fractional shortening were greater following an induced myocardial infarction compared to mice fed a control diet (Zhao et al., 2022), suggesting a benefit to cardiac health.

Only recently has the interaction between diet and cardiac health been evaluated in dogs. In addition, despite the research to support the feeding of high protein and/or high fiber diets in obese or diabetic dogs to reduce weight and improve glucose handling (Diez et al., 2002; Graham et al., 2002; Phungviwatnikul et al., 2022), there is a lack of data on how these diets affect healthy dogs. However, one study reported a decrease in left ventricular end systolic volume in dogs fed a commercial high protein diet compared to a commercial moderate protein diet in healthy Beagles after only 7 d of feeding (Reis et al., 2021), suggesting acute cardiac effects of feeding high protein. Thus, the objective of the present study was to assess the cardiometabolic effects of feeding two diets that used the same ingredients but one providing HPLC and the other providing moderate protein, moderate carbohydrate (MPMC), compared to a current commercial MPMC, high fiber, “metabolic” diet (MET) on cardiac structure and function, fasted serum cardiac troponin I concentrations, and postprandial plasma and whole blood amino acid (AA) concentrations, whole blood glucose concentrations and plasma insulin and glucagon concentrations in healthy, adult, large breed dogs. We hypothesized that cardiac function may be improved in dogs fed MET, including a lower fasted troponin, compared to dogs fed HPLC and MPMC, postprandial AA concentrations would be greater in dogs fed HPLC compared to dogs fed MET and MPMC, postprandial glucose and insulin would be lower in dogs fed HPLC and MET compared to dogs fed MPMC and postprandial glucagon would be greater in dogs fed HPLC compared to dogs fed MET and MPMC.

## MATERIALS AND METHODS

### Animals and Housing

All experimental procedures were approved by the University of Guelph’s Animal Care Committee (AUP #4646) and the study took place in April-September 2022. Nine adult male mixed-breed hounds (1.8 ± 0.1 y, 26.1 ± 0.9 kg, body condition score ranging from 4 to 6 on a 9-point scale (Laflamme, 1997) were housed at the Central Animal Facility at the University of Guelph. Dogs were housed in kennels (7.4 m^2^) either individually with nose-to-nose contact or pair-housed (two pairs). The rooms were maintained at 21 °C with a relative humidity of 50% to 60% and a 12 h: 12 h light:dark schedule with lights on from 0700 h to 1900 h. Dogs had unlimited access to rubber and nylon toys and water within each kennel. All dogs were walked outdoors for 20 min, 6 d/wk, unless weather was poor, in which case dogs were exercised in an indoor playroom for 20 min.

### Diets and Study Design

Three experimental diets were used in this study; a commercially available HPLC extruded diet (Ketona Chicken Recipe For Adult Dogs Dry Dog Food, KetoNatural Pet Foods, Salt Lake City, UT), a MPMC diet, extruded in a commercial plant, formulated with the same ingredients as HPLC, and a commercially available MET extruded diet (Metabolic Chicken Flavour Dog Food, Hill’s Prescription Diet, Topeka, KS). The MET diet is intended for weight loss and to improve satiety. The MPMC diet was formulated to meet a similar protein, fat and carbohydrate content as MET (Table 1), but using the same ingredients as HPLC, replacing a portion of the protein sources (chicken and pea) with brown rice to decrease protein and increase carbohydrate content (Table 2). Both HPLC and MPMC had supplemental vitamins, minerals and choline chloride and the MET diet had supplemental vitamins, minerals, DL-methionine, taurine, L-lysine, choline chloride and L-carnitine included in the formulations. Each dog was fed each diet for 42 d in a complete, randomized, replicated 3 × 3 Latin Square design. The two pairs of dogs that were pair-housed received the same diet in each period in the event of coprophagy. The dogs were fed a wash-in diet (S6 Nutram Sound Balanced Wellness Adult, Nutram Pet Products, Elmira, ON) that contained 24% crude protein, 15% crude fat, 5.5% crude fiber and ~47% nitrogen-free extract for 14 d before and between each of the experimental diets.

**Table 1.** Nutrient analysis and energy content of the high protein, low carbohydrate (HPLC), moderate protein, moderate carbohydrate (MPMC) and metabolic (MET) diet on an as-fed basis

	HPLC	MPMC	MET
**Proximate analysis, %**			
Moisture	7.59	8.12	9.93
Crude protein (CP)	52.1	28.7	27.2
Crude fat	19.0	14.5	12.5
Crude Fiber (CF)	3.28	3.04	10.2
Total dietary fiber (TDF)	7.0	7.9	16.0
Insoluble	10.9	8.4	16.5
Soluble	1.1	0.8	1.7
Calculated nitrogen-free extract (NFE^1^)	10.7	40.0	34.2
Ash	7.38	5.63	6.01
**Energy content**			
Calculated metabolizable energy (ME)^2^, kcal/kg	3,811	3,637	3,210
Crude protein (% of ME)	47.8	27.6	29.7
Crude fat (% of ME)	42.4	33.9	33.1
NFE (% of ME)	9.8	38.5	37.3
**Indispensable amino acid (IDAA), % (Ratio to AAFCO)** ^3^			
Arginine	3.25 (6.9)	1.76 (3.8)	1.25 (2.7)
Histidine	1.14 (6.5)	0.57 (3.3)	0.47 (2.7)
Isoleucine	1.91 (5.4)	1.06 (3.0)	0.86 (2.5)
Leucine	3.39 (5.4)	1.79 (2.9)	2.02 (3.3)
Lysine	3.46 (5.9)	1.65 (2.9)	1.40 (2.5)
Methionine	1.09 (3.6)	0.60 (2.0)	0.99 (3.3)
Methionine & Cystine	1.62 (2.7)	0.92 (1.5)	1.32 (2.3)
Phenylalanine	1.85 (4.4)	1.00 (2.4)	1.00 (2.5)
Phenylalanine & Tyrosine	3.16 (4.6)	1.7 (2.5)	1.81 (2.7)
Threonine	1.83 (4.1)	0.93 (2.1)	0.81 (1.9)
Tryptophan	0.53 (3.6)	0.28 (1.9)	0.22 (1.5)
Valine	2.06 (4.6)	1.16 (2.6)	1.00 (2.3)
**Dispensable amino acid (DAA), %**			
Alanine	2.69	1.51	1.53
Aspartic acid	3.91	2.18	1.95
Cystine	0.53	0.32	0.33
Glutamic acid	6.71	3.76	4.24
Glycine	3.23	1.85	1.49
Proline	2.22	1.21	1.41
Serine	1.75	0.86	0.95
Taurine	0.08	0.07	0.08
Tyrosine	1.31	0.70	0.81
**Other**			
Choline chloride, mg/kg	3,020	2,570	2,380
L-carnitine, mg/kg	27.4	20.6	235
**Protein Quality**			
IDAA:CP	0.49	0.47	0.48
DIAAS-like score^4^	337	192	218

^1^NFE = 100-(moisture + protein + fat + crude fiber + ash).

^2^Metabolizable energy = ((8.5 kcal metabolizable energy (ME) x g crude fat) + (3.5 kcal ME × g crude protein) + (3.5 kcal ME × g nitrogen-free extract) × 10).

^3^Ratio to AAFCO = AA in diet (g/100g dry matter) / AAFCO recommendation for adult dog (g/100g dry matter).

^4^Digestible Indispensable Amino Acid Score (DIAAS)-like score = (mg of IDAA / 1 g protein in diet) / (mg of same IDAA / 1 g protein MR) × 100%, using NRC minimum req for adult dog.

**Table 2.** Ingredient inclusion (%) of the high protein, low carbohydrate (HPLC) and moderate protein, moderate carbohydrate (MPMC) diets and ingredient list of the metabolic (MET)^1^ diet

Ingredient Name	HPLC	MPMC
Brown rice	-	55.78
Chicken, spray-dried	46.97	26.32
Whole green peas	8.75	-
Pea protein (50% min)	8.75	-
Ground chicken	7.90	-
Oat fiber	7.28	8.47
Chicken meal	6.25	-
Chicken fat	5.05	-
Flax meal	3.30	3.84
Gelatin (porcine)	2.11	2.45
Animal digest, dry	1.00	-
Calcium carbonate	0.75	1.00
Potassium chloride, 50%	0.45	0.52
Sea salt	0.45	0.52
Choline chloride, 60%	0.19	0.22
Vitamin C, 35%	0.17	0.18
Mineral premix	0.16	0.16
Naturox plus	0.15	0.18
Citric acid, 99.5%	0.15	0.18
Vitamin premix	0.15	0.14
Albion chelate	0.02	0.02

^1^Whole Grain Wheat, Whole Grain Corn, Chicken Meal, Powdered Cellulose, Soybean Meal, Corn Gluten Meal, Dried Beet Pulp, Dried Tomato Pomace, Hydrolyzed Chicken Flavor, Chicken Fat, Flaxseed, Coconut Oil, Lactic Acid, DL-Methionine, L-Lysine, Carrots, Potassium Chloride, Iodized Salt, Lipoic Acid, vitamins (Vitamin E Supplement, L-Ascorbyl-2-Polyphosphate (source of Vitamin C), Niacin Supplement, Thiamine Mononitrate, Vitamin A Supplement, Calcium Pantothenate, Riboflavin Supplement, Biotin, Vitamin B12 Supplement, Pyridoxine Hydrochloride, Folic Acid, Vitamin D3 Supplement), Choline Chloride, minerals (Manganese Sulfate, Ferrous Sulfate, Zinc Oxide, Copper Sulfate, Calcium Iodate, Sodium Selenite), Taurine, L-Carnitine, Mixed Tocopherols for freshness, Natural Flavors, Beta-Carotene.

Dogs were fed 97.5% of their daily caloric requirement based on historical feeding records and the remaining 2.5% were provided by dehydrated beef lung treats (Beef Tendersticks, Crumps’ Naturals, Brampton, ON). Treats were used as a training tool to provide positive interactions throughout the study. Food was mixed with 200 mL of water and offered once daily at 0745 h. Pair-housed dogs were separated during feeding and all dogs consumed all food within 15 min. Dogs were weighed weekly with calories adjusted as needed in order to maintain BW.

### Echocardiogram and Troponin I

Fasted blood (1 mL) was collected via cephalic venipuncture on day 40 of each period into a serum tube (Becton Dickinson Canada Inc., Mississauga, ON) and subsequently analyzed for high sensitivity cardiac troponin I. Samples were allowed to clot for 30 min and then centrifuged at 4 °C at 2,500 × *g* for 15 min. Serum was separated and frozen at -20 °C to be shipped to the Gastrointestinal Laboratory at Texas A&M University (College Station, TX).

Echocardiograms were performed by a board-certified veterinary cardiologist, who was blinded to dietary treatments, at the Ontario Veterinary College Health Sciences Centre (OVC HSC, Guelph, ON) on day 40 of each period. Echocardiograms were performed using a Vivid E90 echocardiographic system (General Electric, Boston, MA, USA) as previously described (Thomas et al., 1993). All dogs were mildly sedated with 0.2 to 0.4 mg/kg IV butorphanol (Butorphanol Tartrate, 2 mg/mL, Torbugesic, Zoetis Inc., Parsippany-Troy Hills, NJ) before each echocardiogram.

Measurements of left ventricular internal diameter (LVIDd and LVIDs) were obtained from right parasternal short-axis M-mode, volume measurements (LVEDV and LVESV) were made using single plane Simpson’s method of discs from a right parasternal long-axis view or left parasternal apical 4-chamber view, and LV length was measured from the right parasternal long-axis view (Dukes-McEwan et al., 2003; Wess et al., 2010). These measurements were used to calculate fractional shortening (FS% = [(LVIDd-LVIDs)/LVIDd] × 100%), ejection fraction (EF% = [(LVEDV—LVESV)/LVEDV] × 100%), cardiac output (CO = (LVEDV-LVESV) × HR), sphericity index (SI = LVlength/LVIDd), LVIDd and LVIDs normalized to BW (LVIDdN = [LVIDd/10]/[BW^0.294^] and LVIDsN = [LVIDs/10]/[BW^0.315^]) and LVEDV and LVESV indexed to body surface area (BSA; LVEDVi = LVEDV/BSA and LVESVi = LVESV/BSA) (Cornell et al., 2004; Wess et al., 2010). The left atrial size was assessed by the left atrium to aorta ratio (LA:Ao) from a right parasternal short-axis view, and LA long-axis diameter from a right parasternal long-axis view (Rishniw & Erb, 2000). The mitral valve peak E-wave velocity (MVE) and peak A-wave velocity (MVA) were measured from the left apical four chamber view via pulsed wave Doppler and the ratio of peak E-wave and A-wave velocities (E: A) was calculated (Schober et al., 2008). All measurements were obtained in triplicate with the mean value used for analysis.

### Meal Response

Dogs were split into two groups and started each experimental feeding period 1 d apart. On day 42 of each period, for each group, a 12 h meal response was performed. Each dogs’ forelimb was shaved and topical anesthetic (EMLA cream [2.5% lidocaine and 2.5% prilocaine], Astra Pharmaceuticals, L.P. Wayne, PA) was applied. Their forelimb was cleaned with 70% alcohol and then 4% chlorhexidine, and 20 G cephalic catheters (Insyte-W 20 G × 1.1, Becton Dickinson Canada Inc., Mississauga, ON) were placed. A 5 mL fasted sample (time 0) was taken immediately after placement for AA, pancreatic hormone and glucose analysis. A three-way stopcock (Cardinal Health Canada, Vaughan, ON) was attached to each catheter and flushed with 0.5 mL of 50 United States Pharmacopeia (USP) units/mL heparinized saline and locked with 0.5 mL of 100 USP units/mL heparinized saline (Sandoz Canada Inc., Boucherville, QC). Once catheters were placed, dogs were fed their daily food provision starting at approximately 0700 h. Each dog was fed the same amount of calories for the meal challenge in all three periods on the day, even if their calories had been adjusted previously to maintain BW. Immediately after the dog started eating, the timer was started. If dogs did not consume all food within 60 min, the dog was removed from the meal response and only a fasted sample was analyzed. Blood (3.5 mL) was collected into sodium heparin tubes (Becton Dickinson Canada Inc.) at 15, 30, 60, 90, 120, 180, 240, 300 and 360 min post meal for AA analysis. Blood (1 mL) was collected into ethylenediaminetetraacetic acid (EDTA) tubes (Becton Dickinson Canada Inc.) at 15, 30, 60, 90, 120, 180, 240, 300, 360, 420, 480, 540, 600, 660 and 720 min post meal for pancreatic hormone analysis. Dipeptidyl Peptidase-IV (DPP-IV) inhibitor (Millipore Sigma, Billerica, MA, USA), protease inhibitor (Sigma-Aldrich, St. Louis, MO, USA), and Pefabloc SC inhibitor (Sigma-Aldrich, St. Louis, MO, USA) were added to the EDTA tubes, according to the manufacturer, to prevent degradation of hormones. Finally, one drop of whole blood was collected at 5, 10, 15, 20, 25, 30, 60, 90, 120, 180, 240, 300, 360, 420, 480, 540, 600, 660 and 720 min post meal to measure glucose. Whole blood glucose was measured directly after collection using a portable glucose meter, previously validated for use in dogs (Kang et al., 2016; AlphaTRAK 2, Abbott Laboratories, North Chicago, IL). After every sample was taken, the catheter was flushed with 0.5 mL of 50 USP units/mL heparinized saline and locked with 0.5 mL of 100 USP units/mL heparinized saline via the same port on the three-way stopcock, therefore no blood was discarded. For each AA sample, a 0.5 mL aliquot of whole blood was separated and stored on ice. The remaining blood, along with the blood for pancreatic hormone analysis was centrifuged at 4 °C at 1,500 × *g* for 15 min. Plasma was separated and stored on ice. At the end of each sampling day, all samples were moved to a −80 °C freezer until analysis.

### Plasma and Whole Blood AA Analysis

Plasma and whole blood free AA concentrations were analyzed using ultra-performance liquid chromatography (UPLC; adapted from Bidlingmeyer et al., 1984; Waters Corporation, Milford, MA). Before analysis, whole blood was frozen at −80 °C and thawed twice to lyse the red blood cells. In short, 100 μL of 10% sulfosalicylic acid (Sigma-Aldrich, St. Louis, MO) was added to 100 μL of plasma or whole blood to deproteinate. Samples were centrifuged at 14,000 × *g* for 5 min. Amino acid standards and deproteinized samples were derivatized using an AccQ-Tag Ultra derivatization kit (Waters Corporation). The derivatized AAs (1 μL injection) were separated by an AccQ-Tag Ultra RP Column (2.1 × 100 mm, 1.7 μm; Waters Corporation) that was maintained at 55 °C and were detected by UV absorbance (260 nm). Amino acid peak areas were compared with known standards and analyzed with Waters Empower 2 Software (Waters Corporation).

Total plasma homocysteine (hCys), cysteine, cysteinyl-glycine (Cys-Gly) and glutathione (GSH) were analyzed using UPLC using a modified method described in Banton et al. (2021). In short, 30 μL of the reducing agent, tris(2-carboxyethyl)phosphine (Sigma-Aldrich) in 1X phosphate-buffered saline (Thermo Fisher Scientific) was added to 75 μL of plasma and 75 μL of the internal standard, N-(2-Mercaptopropionyl)glycine (2-MPG; Sigma-Aldrich) in 0.1 M K-borate (pH 9.5) + 2 mM EDTA. Samples were placed in the fridge for 30 min and then 125 μL of the derivatizing agent, 70% perchloric acid (Sigma-Aldrich), was added. Samples sat at room temperature for 10 min and were then centrifuged at 14,000 rpm for 5 min. In a light-sensitive centrifuge tube, 30 μL of supernatant was added to 60 μL of 2 M K-borate (pH 10.5) + 5 mM EDTA and 30 μL of the fluorescent thiol-specific dye, 7-fluorobenzofurazan-4-sulfonic acid ammonium salt (Sigma-Aldrich) in 0.1 M K-borate (pH 9.5) + 2 mM EDTA. Samples were incubated in a water bath for 60 min that was maintained at 60 °C and then immediately put on ice for 5 min. Samples were centrifuged again at 14,000 rpm for 1 min and then 100 μL was transferred to a UPLC vial for analysis. The derivatized thiols (1 μL injection) were separated using an Acquity UPLC BEH C18 Column (2.1 × 50 mm, 1.7 μm; Waters Corporation) that was maintained at 24 °C with fluorescence detection at 515 nm emission and 385 nm excitation. Peak areas were compared with known standards and analyzed with Waters Empower 2 Software (Waters Corporation).

### Pancreatic Hormone Analysis

Insulin and glucagon were measured in duplicate using the Milliplex Canine Gut Hormone Magnetic Bead Panel (EMD Millipore Corporation, Billerica, MA, USA) according to the manufacturer’s protocol. The plate was run using a Bio-Plex 200 system with Bio-Plex Data Pro Version 1.3 (Bio-Rad, Mississauga, ON). The quality control samples and standard curves provided in the kit were run according to the manufacturer’s protocol. The coefficient of variation (CV) for each set of duplicates was evaluated and if it was >20%, the results from that sample were discarded.

Homeostatic model assessment of insulin resistance (HOMA-IR) was calculated using HOMA-IR = (insulin (uIU/mL) × glucose (mmol/L))/22.5 and glucose: insulin was calculated using glucose: insulin = glucose (mg/dl)/insulin (uIU/mL) (Radziuk, 2000).

### Statistical Analysis

Echocardiographic and troponin data were analyzed using the proc glimmix function in SAS (v 9.4; SAS Institute Inc., Cary, NC) with dog and period treated as random effects, diet as a fixed effect and heart rate (HR) used as a covariate for the echocardiographic volume and length measurements. Plasma and whole blood AA, plasma insulin and glucagon and whole blood glucose were analyzed as repeated measures using the proc glimmix function with dog and period treated as random effects. The spatial power covariance structure was used and the effect of treatment, time and their interaction was evaluated. The residuals for plasma and whole blood Met were not normally distributed and thus, these data were log transformed for analysis.

Incremental area under the curve (iAUC = area of positive peaks) and net area under the curve (NetAUC = area of positive peaks – area of negative peaks) was calculated using GraphPad Prism (Version 10.2.0) for plasma insulin and glucagon, whole blood glucose, HOMA-IR and glucose: insulin. Incremental AUC and NetAUC were analyzed using the proc glimmix function in SAS with dog and period treated as random effects and diet as a fixed effect. For all outcomes, model assumptions were assessed through residual analysis and if assumptions were violated, a log-transformation was performed. Means were separated using the Tukey-Kramer adjustment and significance was declared at P ≤ 0.05 and trends at 0.05 < P ≤ 0.10.

## RESULTS

### Body Weight and Food Intake

Despite feeding to maintain BW, there were treatment × time interaction effects for BW (kg), food intake (g/d), and calorie intake (kcal/d, P < 0.05, Table 3). Body weight was greater in dogs fed HPLC than dogs fed MET but dogs fed MPMC were similar to both at week 5 and 6. Food intake was greater in dogs fed MET than dogs fed HPLC and MPMC across all weeks. Finally, metabolizable energy (ME) intake, from calculated ME content according to the Modified Atwater equation required by the Association of American Feed Control Officials (AAFCO; Beckman, 2023), was greater in dogs fed MET than dogs fed MPMC but both were similar to dogs fed HPLC at week 5. Metabolizable energy was greater in dogs fed MET than dogs fed HPLC and MPMC at week 6.

**Table 3.** Effect of diet (high protein, low carbohydrate (HPLC), moderate protein, moderate carbohydrate (MPMC) and metabolic (MET)) on body weight, food intake and calorie intake of dogs (n = 9) over 6 wk

Trt	Week						SEM	P-value		
	1	2	3	4	5	6		Trt	Week	Trt× Week
	Body weight, kg									
HPLC	26.4	26.6	26.7	26.7	26.9^a^	26.9^a^	0.8	0.058	0.082	**0.023**
MPMC	26.4	26.5	26.6	26.6	26.7^ab^	26.6^ab^				
MET	26.6	26.5	26.5	26.4	26.4^b^	26.3^b^				
	Food intake, g/d									
HPLC	404.5^b^	398.3^b^	392.7^b^	392.8^b^	378.8^b^	362.3^b^	25.2	**<0.001**	0.223	**<0.001**
MPMC	407.7^b^	401.4^b^	396.8^b^	388.9^b^	381.5^b^	377.2^b^				
MET	449.5^a^	451.7^a^	454.1^a^	460.8^a^	475.1^a^	482.2^a^				
	Calorie intake (ME), kcal/d									
HPLC	1,541.6	1,518.2	1,496.6	1,497.2	1,443.8^ab^	1,380.9^b^	82.2	0.412	0.101	**<0.001**
MPMC	1,482.8	1,460.0	1,443.2	1,414.7	1,387.8^b^	1,372.0^b^				
MET	1,442.8	1,449.9	1,457.7	1,479.4	1,525.1^a^	1,547.8^a^				

^a,b,c^Different letters within the same column indicate statistical significance (P < 0.05)

### Echocardiogram and Serum Troponin I

All parameters measured on the echocardiogram remained within a healthy reference range for 20-30kg dogs (Cornell et al., 2004; Wess et al., 2021, Table 4). Dogs fed MET tended to have a larger LVIDs compared to dogs fed MPMC, but dogs fed HPLC were similar to both (P = 0.062). This trend became significant when normalized to BW (LVIDsN, P = 0.045). Serum cardiac troponin I was different among treatments (P = 0.027) where dogs fed MET (19.8 ± 3.4 pg/mL) had lower concentrations than dogs fed MPMC (30.5 ± 3.4 pg/mL) but dogs fed HPLC (20.5 ± 3.4 pg/mL) were similar to both.

**Table 4.** Effect of diet (high protein, low carbohydrate (HPLC), moderate protein, moderate carbohydrate (MPMC) and metabolic (MET)) on echocardiographic parameters in dogs (n = 9) on day 40 of feeding

	Trt				
Parameter, unit	HPLC	MPMC	MET	SEM	P-value
HR, bpm	86.0	84.0	90.0	6.0	0.706
LAMajor, mm	42.2	41.8	42.2	0.9	0.623
LA:Ao	1.2	1.3	1.3	0.03	0.354
LV length, mm	76.4	76.8	76.8	1.5	0.864
LVIDd, mm	44.3	42.7	44.6	1.5	0.425
LVIDdN,	1.7	1.6	1.7	0.05	0.388
LVIDs, mm	31.4	30.5	33.0	1.3	0.062
LVIDsN^1^	1.19^ab^	1.16^b^	1.26^a^	0.05	**0.045**
LVEDV, mL	84.8	84.9	83.1	4.4	0.629
LVEDVi, mL/m^2^	93.3	93.9	93.0	4.1	0.926
LVESV, mL	39.9	41.5	42.0	3.1	0.278
LVESVi, mL/m^2^	43.8	45.8	46.8	2.9	0.145
FS, %	29.3	28.6	25.9	1.5	0.196
EF, %	52.9	51.7	49.7	2.1	0.186
MVE, m/s	0.68	0.66	0.69	0.03	0.878
MVA, m/s	0.48	0.40	0.48	0.04	0.115
E:A, m/s	1.55	1.79	1.58	0.1	0.210
CO, L/min	3.9	3.6	3.8	0.4	0.751
SI	1.7	1.8	1.7	0.06	0.411

^1^Two decimals presented in order to show difference in lsmean.

### Plasma and Whole Blood AAs

Only two dogs were removed from the meal challenge, one on MET who did not consume his food within 60 min and one on HPLC who was receiving a short bout of pain medication for reasons unrelated to the study. All dogs consumed all food within 15 mins on every meal challenge, except one dog who ate within 60 min on each meal challenge.

All plasma AAs, with the exception of total cysteinyl-glycine (cys-gly), cysteine, hCys, GSH, cystine and Tau had a treatment × time interaction effect as shown in Table 5 (P < 0.05). In general, the plasma AAs that remained elevated over time in dogs fed HPLC compared to the other treatments were Ile, Leu, Lys, Thr, Tyr, Val and the total indispensable amino acids (IDAAs). In dogs fed MET, plasma Gln and Met generally remained elevated over time compared to the other treatments. Interestingly, several AAs were significantly different among treatments at fasting. Plasma Ala, Pro and Trp were greater in dogs fed MET compared to dogs fed HPLC but MPMC was similar to both. Plasma Glu and Met were greater in dogs fed MET and MPMC compared to dogs fed HPLC. Plasma Lys was greater in dogs fed HPLC compared to dogs fed MPMC but MET was similar to both. Plasma cystine, Tau, total hCys and total cys-gly had significant treatment effects (P < 0.05). Dogs fed MET (28 ± 1 µmol/L) and HPLC (28 ± 1 µmol/L) had greater cystine concentrations than dogs fed MPMC (26 ± 1 µmol/L). Dogs fed MET (152 ± 7 µmol/L) had greater Tau concentrations compared to dogs fed HPLC (121 ± 7 µmol/L) who had greater concentrations than dogs fed MPMC (94 ± 6 µmol/L). Dogs fed MET (12 ± 1 µmol/L) had greater total hCys concentrations than dogs fed MPMC (9 ± 1 µmol/L) who had greater concentrations than dogs fed HPLC (7 ± 1 µmol/L). Dogs fed MPMC (7 ± 0.3 µmol/L) had greater total cys-gly concentrations than dogs fed MET (6 ± 0.3 µmol/L) and HPLC (6 ± 0.4 µmol/L). In addition, plasma cystine, Tau, total cysteine and total cys-gly had significant time effects (P < 0.05). Cystine concentrations were greater from 60 to 120 min compared to fasted. Taurine concentrations were lower at 15 and 30 min compared to fasted. Total cysteine concentrations were less than fasted from 15 to 60 min post meal but greater than fasted from 180 to 360 min post meal. Total cys-gly concentrations were less than fasted at 15 min post meal and significantly greater than fasted at 360 min post meal.

**Table 5.** Effect of diet (high protein, low carbohydrate (HPLC), moderate protein, moderate carbohydrate (MPMC) and metabolic (MET)) on plasma amino acid (AA) concentration (µmol/L) over time in dogs (n = 9) at the end of 6 wk

		Time (min)											P-value		
AA	Trt	0	15	30	60	90	120	180	240	300	360	SEM	Trt	Time	Trt × Time
Ala	HPLC	361^b^	330^b^	361^b^	486	482	458	434	432	427	462^a^	24	0.252	**<0.001**	**0.001**
	MPMC	410^ab^	422^a^	423^a^	447	469	457	414	398	388	393^b^	22			
	MET	446^a^	414^a^	447^a^	491	476	464	429	440	436	449^ab^	24			
Arg	HPLC	122	118^ab^	144	280^a^	299^a^	254^a^	203^a^	171^a^	149^a^	145^ab^	12	**<0.001**	**<0.001**	**<0.001**
	MPMC	134	130^a^	134	163^b^	178^b^	169^b^	152^b^	147^ab^	151^a^	154^a^	11			
	MET	109	100^b^	118	146^b^	144^c^	135^c^	118^c^	123^b^	116^b^	118^b^	12			
Asn	HPLC	56	52	64	131^a^	139^a^	131^a^	111^a^	108^a^	102^a^	116^a^	7	**<0.001**	**<0.001**	**<0.001**
	MPMC	49	47	49	66^c^	80^b^	80^b^	76^b^	76^b^	79^b^	82^b^	6			
	MET	60	52	63	89^b^	93^b^	94^b^	89^b^	97^a^	96^ab^	104^a^	7			
Asp	HPLC	9	6^b^	7	12	17^a^	18^a^	22^a^	23^a^	23^a^	26^a^	2	**<0.001**	**<0.001**	**<0.001**
	MPMC	9	13^a^	8	9	11^b^	12^b^	13^b^	14^b^	15^b^	15^c^	2			
	MET	10	7^b^	8	11	13^ab^	15^ab^	16^b^	19^a^	20^a^	21^b^	2			
Total Cys-Gly	HPLC	7	7	7	7	6	6	7	6	6	6	1	**0.004**	**0.002**	0.963
	MPMC	7	8	8	8	7	7	7	7	7	6	1			
	MET	7	7	7	7	6	7	6	6	6	5	1			
Total cysteine	HPLC	88	91	91	93	88	85	84	76	73	79	4	0.131	**<0.001**	0.410
	MPMC	84	89	86	89	85	84	80	82	79	74	4			
	MET	83	94	90	86	83	84	77	75	72	72	4			
Cystine	HPLC	25	26	29	30	31	29	27	28	27	28	2	**0.006**	**<0.001**	0.851
	MPMC	25	26	26	27	28	30	26	26	26	26	2			
	MET	26	28	29	30	32	29	27	26	27	28	2			
Gln	HPLC	710	682	674	679	643	611^b^	601^b^	620^b^	604^b^	615^b^	24	**0.004**	**0.040**	**0.031**
	MPMC	668	661	659	654	664	658^ab^	645^ab^	640^b^	649^b^	652^b^	23			
	MET	678	664	669	704	700	699^a^	677^a^	726^a^	731^a^	750^a^	24			
Glu	HPLC	58^b^	49^b^	51^b^	64	75	77	87^a^	86	85	96^a^	5	0.593	**<0.001**	**<0.001**
	MPMC	72^a^	68^a^	66^a^	69	75	77	77^ab^	79	77	75^b^	5			
	MET	73^a^	64^a^	64^a^	69	72	72	72^b^	75	74	72^b^	5			
Gly	HPLC	280	247	284	451^a^	442^a^	392^a^	328^a^	330^a^	319^a^	346^a^	15	**<0.001**	**<0.001**	**<0.001**
	MPMC	312	287	296	340^b^	374^b^	360^a^	340^a^	328^a^	342^a^	363^a^	15			
	MET	299	261	283	313^b^	305^c^	282^b^	257^b^	268^b^	271^b^	283^b^	15			
Total GSH	HPLC	12	20	17	15	13	12	17	10	16	10	5	0.854	0.372	0.989
	MPMC	15	17	16	16	13	16	15	16	17	13	5			
	MET	17	17	11	17	15	17	14	16	18	11	5			
Total hCys	HPLC	7	8	8	8	7	7	7	6	6	7	1	**<0.001**	0.367	0.622
	MPMC	9	9	9	9	8	8	8	9	9	8	1			
	MET	10	14	12	11	11	12	12	13	13	14	1			
His	HPLC	92	84	86	112^a^	112	115	109	115	113	120	3	0.776	**<0.001**	**0.004**
	MPMC	99	93	94	100^b^	106	110	110	111	113	113	3			
	MET	99	90	95	103^ab^	106	109	106	119	119	120	3			
Ile	HPLC	53	49	60	149^a^	199^a^	221^a^	231^a^	221^a^	217^a^	247^a^	12	**<0.001**	**<0.001**	**<0.001**
	MPMC	50	49	48	71^b^	96^b^	103^b^	97^b^	104^b^	100^b^	104^b^	12			
	MET	52	47	53	80^b^	87^b^	95^b^	91^b^	97^b^	89^b^	90^b^	12			
Leu	HPLC	116	100	116	255^a^	338^a^	373^a^	388^a^	372^a^	366^a^	417^a^	21	**<0.001**	**<0.001**	**<0.001**
	MPMC	102	97	92	125^c^	164^b^	174^c^	164^c^	177^c^	171^c^	177^b^	20			
	MET	115	100	115	185^b^	211^b^	236^b^	228^b^	244^b^	225^b^	226^b^	21			
Lys	HPLC	179^a^	160	197^a^	378^a^	410^a^	375^a^	329^a^	284^a^	267^a^	281^a^	14	**<0.001**	**<0.001**	**<0.001**
	MPMC	137^b^	133	138^b^	180^b^	220^b^	219^b^	215^b^	224^b^	223^b^	219^b^	14			
	MET	155^ab^	141	163^ab^	212^b^	219^b^	216^b^	203^b^	215^b^	207^b^	210^b^	14			
Met	HPLC	45^b^	41^b^	44^b^	75^b^	92^b^	97^b^	99^b^	102^b^	102^b^	114^b^	15	**<0.001**	**<0.001**	**<0.001**
	MPMC	58^a^	54^a^	53^b^	60^b^	69^c^	73^c^	73^c^	76^c^	80^c^	84^c^	14			
	MET	64^a^	59^a^	77^a^	118^a^	155^a^	185^a^	210^a^	244^a^	256^a^	277^a^	15			
Phe	HPLC	57	52	56	89^a^	87^a^	90^a^	84^a^	82^a^	77^a^	82^a^	4	**<0.001**	**<0.001**	**<0.001**
	MPMC	54	52	52	59^b^	66^b^	68^b^	63^b^	60^b^	62^b^	63^b^	4			
	MET	58	54	57	68^b^	72^b^	74^b^	71^b^	75^a^	70^ab^	71^b^	4			
Pro	HPLC	130^b^	121	154	320^a^	357^a^	339^a^	311^a^	315^a^	303^ab^	348^a^	20	**<0.001**	**<0.001**	**<0.001**
	MPMC	169^ab^	158	162	212^b^	257^b^	259^b^	251^b^	249^b^	260^b^	279^b^	19			
	MET	189^a^	162	191	280^a^	310^a^	325^a^	313^a^	340^a^	348^a^	370^a^	20			
Ser	HPLC	144	123	135	206^a^	204^a^	188^a^	167	167	162	177	8	**0.005**	**<0.001**	**<0.001**
	MPMC	141	131	134	144^b^	159^b^	155^b^	152	148	156	165	8			
	MET	147	123	133	160^b^	159^b^	152^b^	144	158	158	169	8			
Tau	HPLC	127	103	107	123	121	110	115	122	132	148	10	**<0.001**	**<0.001**	0.463
	MPMC	121	97	97	95	97	91	89	83	88	82	10			
	MET	172	142	144	152	163	158	150	147	147	148	10			
Thr	HPLC	172	157	171	285^a^	333^a^	324^a^	302^a^	284^a^	277^a^	298^a^	17	**<0.001**	**<0.001**	**<0.001**
	MPMC	172	159	158	178^b^	205^b^	204^b^	192^b^	199^b^	200^b^	207^b^	16			
	MET	167	151	157	187^b^	192^b^	191^b^	178^b^	190^b^	191^b^	200^b^	17			
Trp	HPLC	79^b^	84	95^b^	136^a^	149^a^	154^a^	154^a^	143	148^a^	147^a^	6	**0.005**	**<0.001**	**<0.001**
	MPMC	93^ab^	98	100^ab^	118^b^	130^b^	134^b^	128^b^	135	136^ab^	138^ab^	6			
	MET	95^a^	98	111^a^	122^ab^	125^b^	123^b^	123^b^	130	127^b^	126^b^	6			
Tyr	HPLC	45	42	49	96^a^	113^a^	114^a^	104^a^	95^a^	86^a^	89^a^	6	**<0.001**	**<0.001**	**<0.001**
	MPMC	45	44	43	54^b^	64^b^	67^b^	62^b^	59^c^	63^b^	65^b^	6			
	MET	47	43	48	66^b^	72^b^	75^b^	71^b^	73^b^	68^b^	69^b^	6			
Val	HPLC	182	162	178	322^a^	428^a^	482^a^	525^a^	527^a^	524^a^	574^a^	21	**<0.001**	**<0.001**	**<0.001**
	MPMC	160	152	148	186^b^	234^b^	251^b^	250^b^	272^b^	261^b^	269^b^	20			
	MET	158	145	154	198^b^	218^b^	235^b^	233^b^	250^b^	237^b^	236^b^	21			
IDAA	HPLC	1,097	1,007	1,148	2,082^a^	2,447^a^	2,486^a^	2,426^a^	2,304^a^	2,241^a^	2,431^a^	93	**<0.001**	**<0.001**	**<0.001**
	MPMC	1,060	1,017	1,019	1,241^b^	1,470^b^	1,506^b^	1,445^b^	1,504^b^	1,500^b^	1,528^b^	89			
	MET	1,073	986	1,104	1,429^b^	1,540^b^	1,609^b^	1,575^b^	1,702^b^	1,654^b^	1,693^b^	93			
DAA	HPLC	1,948	1,784	1,916	2,601^a^	2,627^a^	2,470	2,309	2,326^ab^	2,272^ab^	2,454^a^	78	**0.005**	**<0.001**	**<0.001**
	MPMC	2,020	1,954	1,962	2,117^b^	2,276^b^	2,245	2,146	2,099^b^	2,141^b^	2,165^b^	73			
	MET	2,146	1,962	2,082	2,371^a^	2,401^ab^	2,372	2,254	2,379^a^	2,386^a^	2,473^a^	78			

^a,b,c^Different letters within the same row indicate statistical significance (P < 0.05).

All whole blood AAs, with the exception of cystine, Ser and Tau had a treatment× time effect (Table 6, P < 0.05). In general, the whole blood AAs that remained elevated over time in dogs fed HPLC compared to the other treatments were the same ones as in plasma [Ile, Leu, Lys, Thr, Tyr, Val and the total indispensable amino acids (IDAAs)]. In dogs fed MET, whole blood Gln and Met generally remained elevated over time compared to the other treatments. Several AAs were significantly different among treatments at fasting. Whole blood Ala, Glu, Met and Pro were greater in dogs fed MET and MPMC compared to dogs fed HPLC. Whole blood Arg was greater in dogs fed MPMC compared to dogs fed MET but HPLC was similar to both. Whole blood His, Trp and total DAA were greater in dogs fed MET compared to dogs fed HPLC, but MPMC was similar to both. Whole blood Lys was greater in dogs fed HPLC compared to dogs fed MPMC, but MET was similar to both. Whole blood taurine and Thr had significant treatment effects (P < 0.05). Dogs fed MET (238 ± 8 µmol/L) had greater Tau concentrations than dogs fed HPLC (210 ± 8 µmol/L) and MPMC (204 ± 8 µmol/L). Dogs fed HPLC (282 ± 34 µmol/L) had greater Thr concentrations compared to dogs fed MPMC (209 ± 34 µmol/L) and MET (194 ± 34 µmol/L). Whole blood cystine, Ser and Thr had significant time effects (P < 0.05). Cystine concentrations were greater than fasted at 60 min post meal and less than fasted at 360 min post meal. Serine concentrations were less than fasted at 15 and 30 min post meal but greater than fasted at 360 min post meal. However, no time point differed from fasted concentrations for Thr.

**Table 6.** Effect of diet (high protein, low carbohydrate (HPLC), moderate protein, moderate carbohydrate (MPMC) and metabolic (MET)) on whole blood amino acid concentration (µmol/L) over time in dogs (n = 9) at the end of 6 wk

AA	Trt	Time (min)										SEM	P-value		
		0	15	30	60	90	120	180	240	300	360		Trt	Time	Trt × Time
Ala	HPLC	309^b^	291^b^	312^b^	399	406	395	381	377	371	397	19	0.154	**<0.001**	**0.006**
	MPMC	372^a^	367^a^	365^ab^	385	395	392	366	359	348	348	18			
	MET	404^a^	364^a^	383^a^	419	410	408	390	386	383	398	19			
Arg	HPLC	193^ab^	188^a^	204^a^	267^a^	270^a^	257^a^	240^a^	219^a^	210^a^	213^a^	8	**<0.001**	**<0.001**	**<0.001**
	MPMC	200^a^	197^a^	197^a^	212^b^	219^b^	211^b^	203^b^	205^a^	208^a^	205^a^	8			
	MET	180^b^	167^b^	177^b^	192^c^	187^c^	188^c^	182^c^	182^b^	174^b^	180^b^	8			
Asn	MET	41	36	41	54^b^	55^b^	58^b^	57^b^	62^a^	61^ab^	67^ab^	5	**<0.001**	**<0.001**	**<0.001**
	HPLC	35	34	41	76^a^	79^a^	77^a^	69^a^	71^a^	65^a^	74^a^	5			
	MPMC	35	35	35	44^c^	50^b^	51^b^	51^b^	51^b^	54^b^	57^b^	5			
Asp	HPLC	36	33	35	40	46^a^	45^a^	47^a^	45	48^a^	51^a^	3	**0.002**	**<0.001**	**0.025**
	MPMC	38	37	35	38	39^b^	38^b^	39^b^	42	41^b^	41^b^	2			
	MET	39	37	37	41	42^ab^	45^a^	48^a^	48	49^a^	55^a^	3			
Cystine	HPLC	5	5	6	7	5	5	4	5	5	4	1	0.545	**0.001**	0.644
	MPMC	5	6	6	6	5	6	5	5	5	5	1			
	MET	6	6	6	5	5	5	5	5	5	5	1			
Gln	HPLC	612	601	605	615	588	569	564^b^	564^b^	545^b^	559^b^	18	**0.001**	0.144	**0.036**
	MPMC	574	584	571	586	579	577	571^b^	580^b^	583^b^	587^b^	17			
	MET	606	582	592	620	607	615	618^a^	631^a^	630^a^	656^a^	18			
Glu	HPLC	101^b^	98^b^	99^b^	103^b^	108	109	121	119	122	130^a^	5	**0.001**	**<0.001**	**<0.001**
	MPMC	117^a^	116^a^	115^a^	116^a^	115	116	117	122	120	116^b^	5			
	MET	122^a^	112^a^	111^a^	113^a^	113	116	119	120	118	123^ab^	5			
Gly	HPLC	239	219	248	364^a^	369^a^	338^a^	291^a^	288^a^	283^a^	303^a^	13	**<0.001**	**<0.001**	**<0.001**
	MPMC	273	251	256	287^b^	311^b^	309^a^	293^a^	294^a^	304^a^	310^a^	13			
	MET	264	233	241	263^b^	258^c^	251^b^	233^b^	234^b^	240^b^	255^b^	13			
His	HPLC	98^b^	92^b^	98	111^a^	114	114	115	118	117	122	3	0.277	**<0.001**	**0.016**
	MPMC	105^ab^	102^a^	101	103^b^	107	107	110	112	117	115	3			
	MET	108^a^	98^ab^	101	106^ab^	106	112	111	118	117	120	3			
Ile	HPLC	51	47	54	109^a^	145^a^	167^a^	185^a^	182^a^	183^a^	203^a^	8	**<0.001**	**<0.001**	**<0.001**
	MPMC	50	48	47	61^b^	78^b^	85^b^	85^b^	95^b^	91^b^	94^b^	8			
	MET	53	47	50	68^b^	73^b^	82^b^	85^b^	88^b^	83^b^	85^b^	8			
Leu	HPLC	100	90	103	215^a^	282^a^	309^a^	323^a^	310^a^	304^a^	349^a^	16	**<0.001**	**<0.001**	**<0.001**
	MPMC	93	89	86	112^c^	146^b^	151^c^	145^c^	156^c^	151^c^	157^c^	16			
	MET	109	93	104	163^b^	178^b^	205^b^	209^b^	216^b^	200^b^	202^b^	16			
Lys	HPLC	314^a^	296^a^	320^a^	413^a^	431^a^	411^a^	397^a^	369^a^	368^a^	381^a^	10	**<0.001**	**<0.001**	**<0.001**
	MPMC	263^b^	255^b^	253^b^	274^c^	294^b^	289^b^	292^b^	306^b^	300^b^	302^b^	10			
	MET	280^b^	259^b^	272^b^	300^b^	301^b^	304^b^	304^b^	305^b^	299^b^	308^b^	10			
Met	HPLC	36^b^	34^b^	37^b^	58^b^	70^b^	74^b^	76^b^	78^b^	78^b^	86^b^	11	**<0.001**	**<0.001**	**<0.001**
	MPMC	47^a^	44^a^	43^b^	47^c^	54^c^	56^c^	57^c^	60^c^	63^c^	65^c^	10			
	MET	52^a^	48^a^	60^a^	92^a^	114^a^	141^a^	163^a^	183^a^	191^a^	206^a^	10			
Phe	HPLC	50	47	50	67^a^	74^a^	78^a^	76^a^	72^a^	69^a^	73^a^	3	**<0.001**	**<0.001**	**<0.001**
	MPMC	49	47	47	52^c^	57^b^	59^c^	57^c^	57^b^	58^b^	57^c^	3			
	MET	54	49	52	59^b^	62^b^	66^b^	66^b^	67^a^	64^ab^	65^b^	3			
Pro	HPLC	128^b^	121	141	244^a^	278^a^	280^a^	272^a^	274^a^	270^ab^	296^a^	16	**0.001**	**<0.001**	**<0.001**
	MPMC	168^a^	152	154	184^b^	215^b^	221^b^	219^b^	226^b^	236^b^	244^b^	16			
	MET	184^a^	157	171	227^a^	249^ab^	270^a^	276^a^	292^a^	299^a^	319^a^	16			
Ser	HPLC	178	170	170	214	207	204	199	199	184	200	11	0.394	**<0.001**	0.184
	MPMC	183	178	167	177	187	190	179	186	191	199	10			
	MET	183	160	162	181	175	180	186	190	192	209	11			
Tau	HPLC	201	191	198	213	209	208	214	216	222	230	11	**<0.001**	0.183	0.195
	MPMC	203	206	202	213	200	211	203	201	211	191	10			
	MET	244	226	231	235	240	246	248	239	236	242	11			
Thr	HPLC	240	185	228	272	331	300	295	318	321	327	40	**<0.001**	**0.015**	0.119
	MPMC	212	196	201	187	197	221	176	240	241	224	39			
	MET	200	165	193	223	196	205	194	187	160	221	40			
Trp	HPLC	34^b^	38	42	58^a^	60^a^	62^a^	63^a^	61	60	62	3	**0.002**	**<0.001**	**<0.001**
	MPMC	38^ab^	41	42	51^b^	53^b^	56^b^	53^b^	58	60	57	3			
	MET	42^a^	41	45	51^b^	51^b^	53^b^	54^b^	56	56	56	3			
Tyr	HPLC	75	73	77	100^a^	109^a^	110^a^	109^a^	103^a^	102^a^	105^a^	4	**<0.001**	**<0.001**	**<0.001**
	MPMC	75	73	73	77^b^	83^b^	83^b^	80^c^	82^c^	86^b^	86^b^	4			
	MET	79	74	78	87^b^	87^b^	91^b^	92^b^	93^b^	91^b^	94^b^	4			
Val	HPLC	174	157	168	258^a^	328^a^	374^a^	423^a^	428^a^	437^a^	472^a^	17	**<0.001**	**<0.001**	**<0.001**
	MPMC	158	153	144	169^b^	199^b^	213^b^	220^b^	245^b^	238^b^	244^b^	16			
	MET	158	141	147	176^b^	187^b^	207^b^	214^b^	225^b^	219^b^	222^b^	17			
IDAA	HPLC	1,048	987	1,075	1,554^a^	1,773^a^	1,846^a^	1,898^a^	1,837^a^	1,827^a^	1,964^a^	58	**<0.001**	**<0.001**	**<0.001**
	MPMC	1,003	975	960	1,082^b^	1,207^b^	1,228^b^	1,222^c^	1,295^b^	1,286^b^	1,296^c^	55			
	MET	1,037	943	1,010	1,212^b^	1,266^b^	1,360^b^	1,396^b^	1,450^b^	1,413^b^	1,454^b^	58			
DAA	HPLC	1,919^b^	1,838	1,931	2,375^a^	2,404^a^	2,340	2,271	2,259	2,215	2,349^a^	66	**0.018**	**<0.001**	**0.005**
	MPMC	2,041^ab^	2,005	1,977	2,111^b^	2,178^b^	2,194	2,123	2,148	2,179	2,156^b^	63			
	MET	2,172^a^	1,986	2,053	2,245^ab^	2,239^ab^	2,283	2,268	2,297	2,302	2,420^a^	66			

^a,b,c^Different letters within the same row indicate statistical significance (P < 0.05).

**Figure 1.**
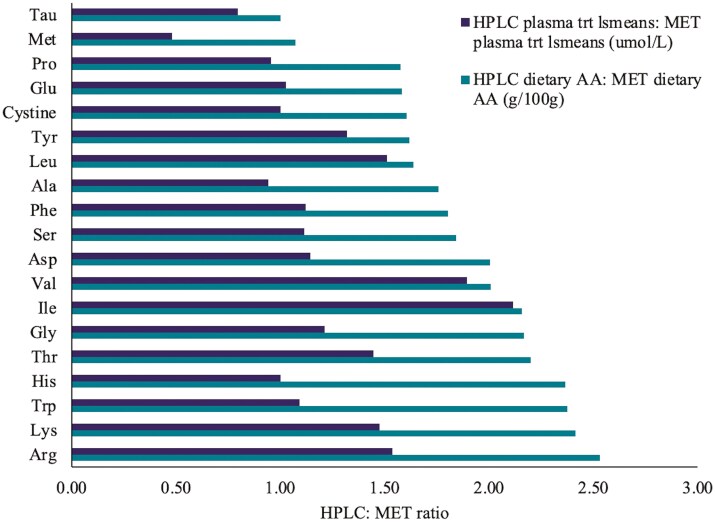
Comparison of the ratio of dietary amino acid (AA) content (g/100g) and plasma AA (umol/L) content across time in the high protein, low carbohydrate (HPLC): metabolic (MET) diet. Plasma values are treatment lsmeans.

The ratio of dietary AAs between HPLC and MET and the corresponding ratio of plasma concentrations across time points between HPLC and MET are shown in Figure 1. Only plasma Leu, Ile and Val had similar dietary and plasma ratios. All other AA had smaller plasma ratios compared to dietary ratios.

### Glucose, Insulin and Glucagon Concentrations

**Figure 2.**
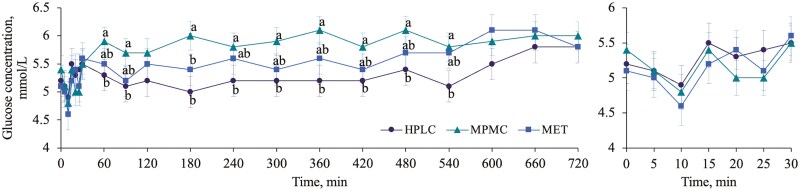
Treatment by time interaction effect for whole blood glucose concentrations (mmol/L) across diet (high protein, low carbohydrate (HPLC), moderate protein, moderate carbohydrate (MPMC) and metabolic (MET)) after 6 wk of feeding in dogs (n = 9). Left panel: complete time course. Right panel: 0-30 min showing each sample taken 5 min apart.

**Figure 3.**
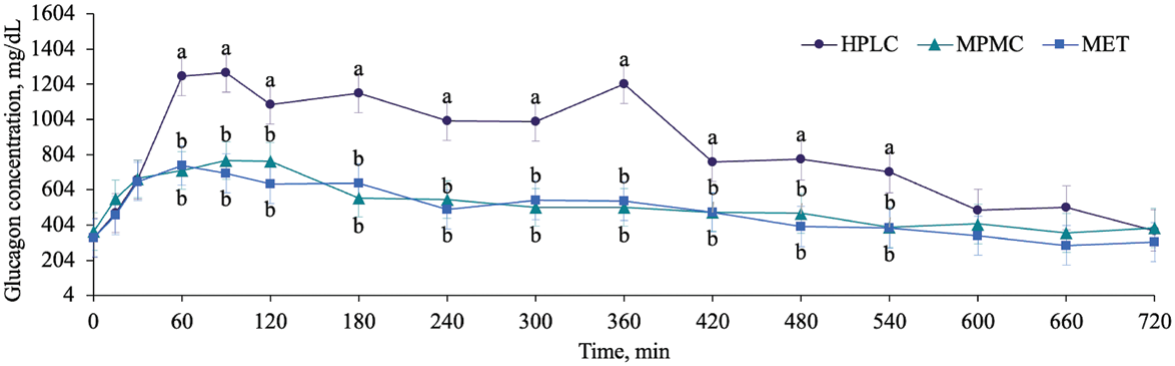
Treatment by time interaction effect for plasma glucagon concentrations (mg/dL) across diet (high protein, low carbohydrate (HPLC), moderate protein, moderate carbohydrate (MPMC) and metabolic (MET)) after 6 wk of feeding in dogs (n = 9).

Two individual samples for plasma insulin and glucagon from two different dogs at two different time points were removed due to a CV > 20%. All other samples were included. There was a trend for a treatment× time interaction effect for glucose (P = 0.071, Figure 2) and a significant effect for glucagon (P < 0.001, Figure 3) concentrations over time. Dogs fed MPMC had greater glucose concentrations than dogs fed HPLC at 60, 90, 180, 240, 300, 360, 420, 480 and 540 min. Dogs fed HPLC had greater glucagon concentrations than dogs fed MPMC and MET from 60 to 540 min post meal. There was a significant time effect for plasma insulin concentrations (P < 0.001) but no difference among treatments. Plasma insulin was greater from 30 to 480 min post meal compared to fasted concentrations. The HOMA-IR tended to have a treatment effect (P = 0.054) and had a significant time effect (P < 0.001). Dogs fed MPMC (15.5 ± 2.4) had a greater HOMA-IR than dogs fed HPLC (12.9 ± 2.4), but dogs fed MET (13.2 ± 2.4) were similar to both. Additionally, the HOMA-IR was greater from 60 to 480 min post meal compared to fasted concentrations. Although the null hypothesis was rejected in the F-test for a treatment by time effect (P = 0.007), no significant differences were observed between treatments at each time point for glucose:insulin when pairwise comparisons were analyzed using the Tukey–Kramer adjustment.

The netAUC (positive peaks – negative peaks) and iAUC are presented in Table 7. NetAUC tended to be different among treatments (P = 0.057) for glucose, where dogs fed MPMC had a greater netAUC than dogs fed HPLC and dogs fed MET tended to have a greater netAUC than HPLC. Both netAUC and iAUC were significant for plasma glucagon (P < 0.001) where dogs fed HPLC had a greater AUC than dogs fed MPMC and MET. Incremental AUC tended to be different for the glucose: insulin ratio (P = 0.093) where dogs fed MPMC tended to have a greater area than dogs fed HPLC but dogs fed MET were similar to both. Finally, netAUC was significant for the glucose: insulin ratio (P = 0.039) where dogs fed HPLC had a greater AUC than dogs fed MPMC but dogs fed MET were similar to both.

**Table 7.** Effect of diet (high protein, low carbohydrate (HPLC), moderate protein, moderate carbohydrate (MPMC) and metabolic (MET)) on incremental area under the curve (iAUC) and NetAUC for whole blood glucose, plasma glucagon and insulin and HOMA-IR and glucose: insulin over time in dogs (n = 9) at the end of 6 wk

		Trt			
	Parameter	HPLC	MPMC	MET	P-value
Glucose (mmol/L*min)	iAUC	236.9 ± 79.0	397.8 ± 75.5	386.5 ± 74.9	0.103
	NetAUC	129.3 ± 95.0	361.8 ± 90.4	346.7 ± 89.8	0.057
Glucagon (pg/mL*min)	iAUC	346,481 ± 40,073^a^	102,086 ± 38,411^b^	115,168 ± 38,129^b^	**<0.001**
	NetAUC	344,946 ± 39,992^a^	93,697 ± 38,178^b^	111,955 ± 37,900^b^	**<0.001**
Insulin (uIU/mL*min)	iAUC	23,080 ± 5,046	25,190 ± 4,872	22,392 ± 4,844	0.820
	NetAUC	22,887 ± 5,135	24,962 ± 4,956	22,370 ± 4,928	0.844
HOMA-IR	iAUC	5,562 ± 1,476	7,112 ± 1,429	6,179 ± 1,421	0.502
	NetAUC	5,515 ± 1,497	7,059 ± 1,449	6,175 ± 1,442	0.521
Glucose: Insulin	iAUC*	1,691 ± 663	3,229 ± 629	2,048 ± 624	0.093
	NetAUC	−1,032 ± 715^a^	−2,906 ± 692^b^	−1,979 ± 687^ab^	**0.039**

iAUC= Incremental Area Under the Curve (positive peaks).

NetAUC = Area of positive peaks – area of negative peaks.

^*^Negative peaks used for Glucose: Insulin.

## DISCUSSION

In general, the HPLC diet increased postprandial AAs, especially the branched chain AAs (BCAA), increased the postprandial Net and iAUC for plasma glucagon, decreased the NetAUC for the glucose: insulin ratio and tended to decrease the NetAUC for blood glucose compared to two control MPMC diets. This suggests that a HPLC diet can shift intermediary metabolism in young, healthy, adult, lean dogs in contrast to a commercial diet intended for weight loss that employs a number of different mechanisms, including fiber and micronutrients.

As hypothesized, the majority of postprandial plasma and whole blood AAs were greater in dogs fed HPLC, aligning with their higher concentrations in the diet. However, for plasma and whole blood Met, this was not the case, despite the HPLC diet having higher Met concentrations than the other two diets. This is likely because the MET diet was supplemented with DL-methionine, a synthetic AA that is absorbed faster and is considered 100% bioavailable compared to protein bound Met (Weijzen et al., 2022). Despite the HPLC diet having 110% higher Met concentrations than MET, dogs fed MET had a peak Met concentration that was 243% higher than dogs fed HPLC, which may be expected given the faster absorption. This was also the case for all plasma AAs in humans fed free synthetic AAs versus intact whey protein, even when they were provided at the same concentrations (Weijzen et al., 2022). The same study, through the use of isotope tracers, found that the free AA protein source increased the availability of AAs for peripheral tissues, but there was no difference in muscle protein synthesis rate between protein sources (Weijzen et al., 2022). This helps explain the higher hCys concentrations found in dogs fed MET, given that Met is a precursor to hCys. It is important to point out that Weijzen et al. (2022) only used protein sources whereas the present study used complete diets with carbohydrates and fats, which can affect absorption of AAs. For example, carbohydrates can decrease the appearance of BCAAs in the blood when fed in combination with protein and fat (Hagve et al., 2021). In fact, plasma BCAA concentrations have been shown to be a better predictor of the carbohydrate content of the diet than glucose (Hagve et al., 2021).

Interestingly, postprandial plasma and whole blood Gln concentrations were also greater in dogs fed MET compared to dogs fed HPLC and MPMC. More specifically, plasma and whole blood Gln increased after the meal in dogs fed MET but decreased in dogs fed HPLC and MPMC. Blood Gln concentrations are inversely related to dietary protein intake (Matthews & Campbell, 1992; Moundras et al., 1993). Given that the majority of Glu and Gln is catabolized by the gut in first pass metabolism (Stoll et al., 1998) and Gln and Ala are the predominant AAs released from muscle in the post-absorptive state (Elia et al., 1988), it is thought that a higher protein intake leads to a lower output of Gln and Ala from muscle (Forslund et al., 2000). Our results partially support this theory, assuming that our fasted sample is representative of a post-absorptive state, given that both Ala and Glu were lower in dogs fed HPLC than dogs fed MET at time 0. However, it remains unclear as to why dogs fed MPMC had similar postprandial concentrations of Gln to dogs fed HPLC, given that the protein content of MPMC was almost half that of HPLC.

Amino acids can stimulate both insulin and glucagon release from the pancreas, although, their stimulatory capacity is not as strong as glucose (Schmid et al., 1989). As hypothesized, glucagon increased to a greater extent, postprandially, in dogs fed HPLC compared to the other diets. However, there was no effect of diet on plasma insulin concentrations. Previous studies in rats suggest that glucagon responds to the amount of protein present whereas insulin responds to the type of protein present (Tovar et al., 2002). Given that our three diets all had the same main protein sources (chicken and chicken meal) but the HPLC diet had almost double the amount of protein, our findings align with previous work. Humans with diabetes have been shown to have elevated fasted and postprandial glucagon concentrations and delayed glucagon suppression after a glucose tolerance test compared to healthy individuals (Færch et al., 2016). In the present study, the glucagon concentrations were elevated for longer in dogs fed HPLC and returned to fasted values by 420 min, compared to 120 min in dogs fed MET and MPMC. However, the glucagon concentrations were not different between diets at 0 to 30 and 600 to 720 min, suggesting that the dogs fed HPLC were able to suppress the postprandial glucagon within 10 h, as expected for a healthy dog. In addition, glucagon has been shown to play a role in appetite suppression, weight loss and energy expenditure in humans and rats (reviewed in Al-Massadi et al., 2019). Therefore, the prolonged elevations in glucagon may promote weight loss and support additional findings from this study that dogs fed HPLC had greater postprandial energy expenditure compared to dogs fed MPMC (Banton et al., 2025).

An important consideration of the diets used in the present study is that although they all exceeded the AAFCO IDAA recommendations, the ratios of IDAA supply relative to AAFCO varied dramatically between MET and MPMC compared to HPLC. The supply of IDAAs in the diets ranged from 1.5 to 3.3 and 1.5 to 3.8 times the AAFCO recommendations for MET and MPMC, respectively, compared to 2.7 to 6.9 times for HPLC. This suggests that a much larger portion of AAs provided by the HPLC diet needed to be catabolized. A high influx of AAs from dietary intact proteins has been shown to increase deamination in order to prevent hyperaminoacidemia (Bos et al., 2003). Although we did not measure deamination of AAs, we reported much lower ratios of HPLC plasma AA: MET plasma AA compared to the corresponding HPLC dietary AA: MET dietary AA. This is in contrast to Bos et al. (2003) who reported very similar ratios of dietary AA levels and serum dietary N-containing AAs in humans fed 15-N labeled casein versus soy mixed meals. Our dietary AAs were not labeled, but if, as hypothesized, the dietary AA concentrations are the main determinant of the plasma AA concentrations, then the dietary and plasma AA ratios should be similar. However, we only report very similar ratios for the BCAAs and, not surprisingly, the plasma and whole blood BCAAs, Leu, Ile and Val all increased dramatically following a meal in dogs fed HPLC compared to the other diets. This could be due to the fact that the enzyme responsible for deamination of the BCAAs is not influenced by dietary protein intake or hormones, except for in the skeletal muscle (Torres et al., 1998). In addition, this is supportive of previous data that suggests that higher dietary protein leads to greater AA deamination, mentioned above. Recent literature suggests a strong association between plasma BCAAs, insulin resistance and development of type 2 diabetes in humans, however, this depends on a number of other factors such as obesity and inflammation (reviewed in Karusheva et al., 2021; White et al., 2021). This is in contrast to the reported effects of a HPLC diet on lowering plasma glucose and insulin in people with type 2 diabetes (Seino et al., 1983; Gannon & Nuttall, 2004). However, there are numerous factors that need to be considered in terms of disease onset and progression, of which most have been researched in humans, but not dogs.

In a previous study conducted using healthy adult dogs and three diets similar to the ones used in the present study (a HPLC, MPMC and MPMC-high fiber), insulin concentrations were higher in dogs fed MPMC only at 4 and 6 h post meal in a 12 h meal response (Elliott et al., 2012), however, AUC was not calculated. Although we did not have a significant time by treatment effect at any timepoint, it is likely that if the authors had calculated AUC, it would not be different between diets, as was the case in the present study. In the same study, dogs fed the HPLC diet had lower glucose concentrations than one or both other diets at 1, 2, 4, 5 and 6 h post meal. Similarly, we found that dogs fed HPLC had lower glucose concentrations than dogs fed MPMC at 1, 1.5 and 3 to 9 h post meal but similar concentrations to dogs fed MET. Although numerically smaller, dogs fed HPLC only tended to have a lower NetAUC for glucose compared to dogs fed MET. This could be due to multiple factors. First, the variation in glucose values that we report is quite high, likely due to the physiological variation in glucose uptake among dogs. Second, we used the Tukey-Kramer adjustment which is more conservative and was chosen to control Type 1 error. Third, the present study was done in healthy, lean, young dogs who are insulin sensitive and have improved glucose uptake compared to obese dogs (Verkest et al., 2012). It is likely that our results would be more pronounced in obese or diabetic dogs as they would have greater circulating glucose concentrations. However, our results in healthy dogs suggest a trend towards lower blood glucose when fed HPLC. Finally, the MET diet had double the amount of total dietary fiber and 1.5 times the amount of insoluble fiber compared to HPLC. In dogs with diabetes receiving insulin, a diet high in insoluble fiber lowered blood glucose concentrations over time, as well as peak blood glucose concentration (Kimmel at al., 2000). Therefore, the higher fiber in the MET diet likely contributed to the intermediate glucose values observed in dogs fed MET compared to dogs fed HPLC and MPMC.

Despite feeding to maintain BW, dogs fed MET had a higher calculated ME intake and lower BW than dogs fed HPLC and MPMC by week 6. Although the modified Atwater equation used to calculate ME is what is currently recommended by AAFCO, it has been shown to be inaccurate depending on the macro and micronutrient inclusions in the diet (Asaro et al., 2017), and dependent on type of diet (Jewell & Jackson, 2023). Although the differences in BW are not biologically relevant, the higher fiber and L-carnitine content of the MET diet likely led to this disparity, given that both high fiber and L-carnitine can promote weight loss in dogs (Floerchinger et al., 2015). Although not a primary outcome of interest in the present study, it is interesting that the energy intake of the HPLC diet had to be reduced over the 6 wk to maintain BW, given that previous studies in obese humans suggest that HPLC diets can promote weight loss without calorie restriction (Nickols-Richardson et al., 2005; Krebs et al., 2010). However, these dogs were not obese and their calories needed to be reduced in order to prevent weight gain on the HPLC diet. As discussed in Banton et al., (2025), it is likely that the energy density of the HPLC diet was overestimated using the Modified Atwater equation and this lead to the dogs being overfed in the first few weeks, leading to the decrease in calories over the weeks.

All parameters that were measured on echocardiography remained within normal reference ranges for dogs between 20-30 kg in weight. Although the LVIDsN was larger in dogs fed MET compared to dogs fed MPMC, it is not a biologically relevant change and does not align with previous literature that is supportive of both high fiber and L-carnitine being cardioprotective. Using mice as a model for human myocardial infarction (MI), mice fed a high fiber diet for 3 wk had decreased LVIDs following MI compared to mice fed a standard diet (Zhao et al., 2022). Carnitine is found in high concentrations in the heart (Rebouche & Engel, 1983) because it plays a role in generating energy as a cofactor for fatty acid transport into the mitochondria (Gilbert, 1985). Similarly, human patients supplemented with 6 g/d oral L-carnitine had improved left ventricle dilation, as measured by LVESV and LVEDV, after MI compared to those given a placebo, however this was after a year of supplementation (Iliceto et al., 1995). In support of our hypothesis and the cardioprotective role of fiber, we did find that dogs fed MET had lower cardiac troponin I concentrations, a marker of cardiac damage, compared to dogs fed MPMC, although all remained within the reference range. In the only other study to investigate cardiac structure and function in healthy Beagles fed a high protein diet for 7 d, Reis et al. (2021) report a smaller LVESV in dogs fed the commercial high protein diet compared to the commercial moderate protein diet. However, neither value was outside of the healthy reference range for that size of dog and there were no changes in diameter of the left ventricle. In addition, not all protein is equal and the amount of protein provided is not necessarily representative of the AA availability. Therefore, the availability of the AA provided by the two commercial diets used by Reis et al. (2021) may be quite different compared to the HPLC and MPMC diets used in the present study. Taken together, it is unlikely that feeding high protein commercial diets or a high fiber, L-carnitine-supplemented diet to healthy dogs leads to changes in cardiac structure or function in the short-term. Longer feeding trials may be done in order to confirm if this is the case in the long-term.

To the author’s knowledge, this is the first study to investigate the cardiometabolic effects of feeding a HPLC diet compared to a MPMC diet made with the same ingredients and a commercially available MPMC, high fiber, L-carnitine-supplemented diet. The biggest difference between these diets was the appearance of glucagon and AAs in the blood, as hypothesized. Dogs fed the HPLC diet had greater postprandial glucagon and AA concentrations, with the exception of Met and Gln, but especially high concentrations of the BCAAs in relation to the amount provided by the diet. The BCAAs deserve further study in relation to the development/progression of diabetes in dogs. Our results in healthy, lean dogs suggest a trend towards lower blood glucose in dogs fed HPLC compared to dogs fed MPMC and MET and the magnitude of change in glucose concentrations would likely be more evident in obese or diabetic dogs. Overall, no diet led to any major differences in cardiac structure or function but dogs fed MET had lower cardiac troponin I concentrations compared to MPMC. Future studies may investigate similar outcomes in dogs in a clinical setting, whether that be cardiac related or in obese or diabetic dogs to see more pronounced effects of the diets tested here.
